# Antitumor Effect of Some 3d-Metal Complexes of N-Isonicotinoyl-N'-o-Hydroxythiobenzhydrazide

**DOI:** 10.1155/S1565363303000207

**Published:** 2003

**Authors:** Anuraag Shrivastav, Nand K. Singh, Saty B. Singh, Sukh M. Singh, Nisha Singh, Anil Shanker

**Affiliations:** 1 Department of Chemistry Banaras Hindu University Varanasi 221 005 India; 2 School of Biotechnology Banaras Hindu University Varanasi 221 005 India

## Abstract

A new ligand, N-isonicotinoyl-N'-*o*-hydroxythiobenzhydrazide (H_2_Iotbh), forms complexes [Co(Iotbh)(H_2_O)_2_], [M(Iotbh)] [M Ni(II) Cu(II) and Zn(ll)] and [M(Iotbh-H)(H_2_O)_2_] [M Mn(III), Fe(III)], which were characterized by various physico-chemical techniques. DMSO solution of metal complexes was observed to inhibit the growth of tumor in vitro, whereas the ligand did not. In vivo administration of these complexes resulted in prolongation of survival of tumor-bearing mice. Tumor-bearing mice administered with the solution of metal complexes showed reversal of tumor growth associated induction of apoptosis in lymphocytes. The paper discusses the possible mechanisms and therapeutic implications of the H_2_lotbh and its metal complexes in tumor regression and tumor growth associated immunosuppression.


### Antitumor Effect of Some 3d-Metal Complexes of N-Isonicotinoyl-N'-o-Hydroxythiobenzhydrazide

Anuraag Shrivastav, Nand K. Singh* and Saty B. Singh
*Department of Chemistry, Banaras Hindu University, Varanasi-221 005, India.*
Sukh M. Singh, Nisha Singh and Anil Shanker.
*School of Biotechnology, Banaras Hindu University, Varanasi-221 005, India.*

### ABSTRACT

A new ligand, N-isonicotinoyl-N'-*o*-hydroxythiobenzhydrazide ($H_2$Iotbh), forms complexes [Co(Iotbh)($H_2O$)$_2$], [M(Iotbh)] [M = Ni(II) Cu(II) and Zn(II)] and [M(Iotbh-H)($H_2O$)$_2$] [M= Mn(III), Fe(III)], which were characterized by various physico-chemical techniques. DMSO solution of metal complexes was observed to inhibit the growth of tumor *in vitro*, whereas the ligand did not. *In vivo* administration of these complexes resulted in prolongation of survival of tumor-bearing mice. Tumor-bearing mice administered with the solution of metal complexes showed reversal of tumor growth associated induction of apoptosis in lymphocytes. The paper discusses the possible mechanisms and therapeutic implications of the $H_2$Iotbh and its metal complexes in tumor regression and tumor growth associated immunosuppression.

### INTRODUCTION

Transition metal complexes of a few thiohydrazides and their N-substituted derivatives have been reported /1-6/. Hydrazones derived from the condensation of isonicotinic acid hydrazide(INH) with pyridine aldehydes were found to possess better antitubercular activity than INH /7/, and this was attributed to their metal chelating abilities /8-12/. Recently it was confirmed that INH forms a lipid-soluble copper chelate which facilitates cell internalization and it has been approved as a first line antitubercular compound used in DOT programme advocated by WHO /13,14/. Pyridine-2- and -4- carboxaldehyde isonicotinoyl hydrazones (HL) form complexes of the type M(HL)$Cl_2$. The ligands and their Co(II), Ni(II) and Zn(II) complexes were found to display significant antibactrial activity /15/. Cu(II) complex of N-salicyloyl-N'-2-furanthiocarbonyl hydrazine ($H_2$sfth) was found to have square planar geometry around Cu(II). The ligand, $H_2$sfth, showed better antitumor activity than its Cu(II) complex /16/. The metal complexes of 4-hydroxyphenyl thiocarboxy hydrazide and N-salicyloyl-N'-*p*-hydroxybenzthioyl hydrazine were found to be potent antiviral, antifungal, antibacterial and antitumor agents /17,18/. In view of the fact that both isonicotinoyl and thiosalicyloyl

---

* Corresponding author. E-mail: sbsbhu@yahoo.co.in groups were reported to display various biological activities such as antibacterial and antitumor, it appeared worthwhile to synthesize, characterize and study the antitumor and immunomodulatory effect of N-isonicotinoyl-N'-o-hydroxythiobenhydrazide [(4-$C_5H_4N$)C(O)NHNHC(S)2-(HO)$C_6H_4$] and its 3d metal complexes.

### RESULTS AND DISCUSSION

The analytical data (Table 1) correspond to the 1:1 metal-to-ligand stoichiometry and the complexes are coloured. The metal(II) complexes were formed by loss of two protons from the ligand and metal (III) complexes by loss of one additional proton from the hydroxy group of the salicyloyl moiety. Mn(II) complex could not be isolated because it is unstable, but after some time due to aerial oxidation of Mn(II) to Mn(III), the colour of the solution changed from wine red to maroon from which the solid Mn(III) complex could be isolated by the addition of acetonitrile. The water molecules are lost on heating the complexes at 120-150°C, suggesting the coordinate nature of these molecules. Because of steric considerations, all four potential sites cannot be attached to a single metal and therefore, the ligand binds in a polymeric fashion. The complexes are soluble in polar coordinating solvent such as DMSO and melt above 340°C, except the complexes of Co(II), Cu(II) and Zn(II).

### Magnetic properties and electronic spectra

The magnetic moments of Mn(III), Fe(III) and Co(II) complexes suggest their distorted octahedral geometry which is further supported by the UV-Vis spectral data. [Mn(Iotbh-H)($H_2O$)$_2$] exhibits a magnetic moment of 4.9 B.M. and shows two d-d bands at 16850 and 18050 $cm^{-1}$ assigned to the $^5B_{1g} \rightarrow {}^5A_{1g}, {}^5T_{2g}$ transitions, respectively for six coordinate Mn(III). For [Fe(Iotbh-H)($H_2O$)$_2$], a magnetic moment of 5.9 B.M. and an UV-Vis band at 20830 $cm^{-1}$ assigned to d-d or charge-transfer transition clearly suggest a high-spin iron(III) centre. [Co(Iotbh)($H_2O$)$_2$] exhibits a magnetic moment of 5.0 B.M., suggesting a high-spin octahedral geometry around Co(II) which is further supported by the occurrence of electronic spectral bands at 16800 and 19560 $cm^{-1}$ assigned to the $^4T_{1g} \rightarrow {}^4A_{1g}, {}^4T_{1g}$ (P) transitions, respectively. The magnetic moment and UV-Vis spectral data of Cu(II) and Ni(II) complexes suggest their square-planar geometry. [Ni(Iotbh)] is diamagnetic and displays bands at 16500 and 20350 $cm^{-1}$ which may be assigned to the $^1A_{1g} \rightarrow {}^1B_{2g}$ and $^1B_{1g}$ transitions, respectively. [Cu(Iotbh)] exhibits a magnetic moment of 1.78 B.M. and the presence of a broad d-d band at 16835 $cm^{-1}$ assigned to the envelope of $^2B_{1g} \rightarrow {}^2A_{1g}$, $^2B_{2g}$ and $^2E_g$ transitions suggests a square-planar geometry around Cu(II).

**Table 1**

Analytical data and physical properties of $H_2$Iotbh and its metal complexes

| Compound | Colour | Mp (°C) | Yield (%) | Found (Calcd..) % | | | | | $\mu_{eff}$ (BM) |
|---|---|---|---|---|---|---|---|---|---|
| | | | | M | S | C | H | N | |
| $H_2$Iotbh | Yellow | 238 | 60 | - - | 11.0 (11.7) | 57.6 (57.1) | 4.2 (4.0) | 15.5 (15.4) | - - |
| [Mn(Iotbh-H)($H_2O$)$_2$] | Maroon | >340 | 57 | 14.5 (15.2) | 8.1 (8.6) | 43.4 (43.2) | 2.9 (3.3) | 10.9 (11.6) | 4.9 |
| [Fe(Iotbh-H)($H_2O$)$_2$] | Black | >340 | 68 | 16.1 (15.4) | 8.6 (8.8) | 42.7 (43.1) | 2.6 (3.3) | 11.2 (11.6) | 5.9 |
| [Co(Iotbh)($H_2O$)$_2$] | Green | 313 | 60 | 16.9 (16.1) | 9.4 (8.7) | 42.4 (42.6) | 2.7 (3.6) | 11.8 (11.5) | 5.0 |
| [Ni(Iotbh)] | Brown | >340 | 61 | 17.2 (17.8) | 10.2 (9.7) | 47.1 (47.3) | 2.5 (2.7) | 11.9 (12.7) | Dia |
| [Cu(Iotbh)] | Black | 305 | 69 | 18.3 (19.0) | 10.5 (9.6) | 46.9 (46.6) | 2.8 (2.7) | 12.1 (12.6) | 1.78 |
| [Zn(Iotbh)] | Light Green | 295$^d$ | 70 | 18.7 (19.4) | 8.9 (9.5) | 46.7 (46.4) | 2.2 (2.7) | 12.7 (12.5) | Dia |

d = decompose

### I R spectra

The IR spectrum of $H_2$Iotbh shows bands at 3447 and 3202 $cm^{-1}$ assigned to ν(OH) and ν(NH), respectively. The bands occurring at 1674, 1433, 1332, 1060 and 960 $cm^{-1}$ are assigned to ν(C=O), thioamide I [β(NH) + ν(CN)], thioamide II [ν(CN) + β(NH)], ν(N-N) and ν(C=S), respectively. A broad band in the region of 3450-3460 $cm^{-1}$ observed in the spectra of hydrated complexes may be assigned to the ν(OH) of the ligand as well as of water molecules. The bands due to ν(NH), ν(C=O) and ν(C=S) of the ligand are absent in the complexes and in place of these, two new bands appear in the regions of 1605-1620 $cm^{-1}$ and 820-860 $cm^{-1}$ due to ν(C=N) of NCO and ν(C—S) modes, respectively, suggesting that both -NHNH- protons are lost via enolisation and thioenolisation and bonding of the resulting enolic oxygen and thiolato sulfur takes place with the metal ion /19/. The thioamide I, II and ν(N-N) undergo a positve shift of 25, 30 and 40 $cm^{-1}$, respectively, suggesting bonding through thiolato sulfur and hydrazinic nitrogen. [M(Iotbh)] [M = Ni(II), Cu(II) and Zn(II)] exhibit bands for ν(OH) in the region of 3435- 3456 $cm^{-1}$. [M(Iotbh-H)($H_2O$)$_2$] [M = Mn(III) and Fe(III)] show the absence of bands due to ν(OH) (phenolic), ν(NH), ν(C=O) and ν(C=S) which suggests loss of both hydrazinic protons (via enolisatisation and thioenolisation) and of phenolic proton.This

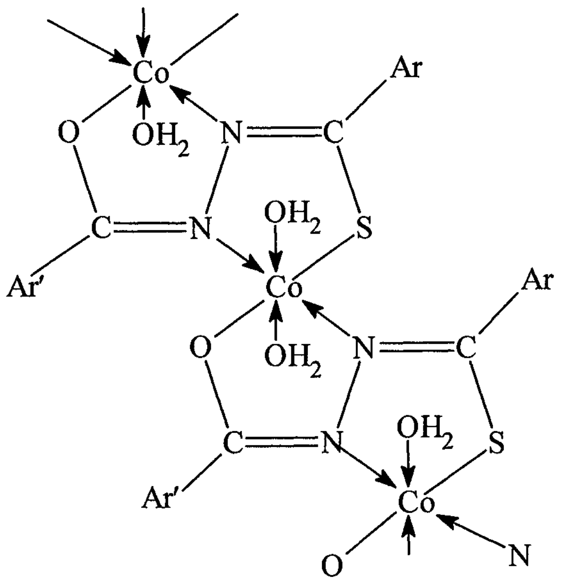

$[Co(Iotbh)(H_2O)_2]$

$Ar = 2\text{-}(HO)C_6H_4$, $Ar' = 4\text{-}C_5H_4N$

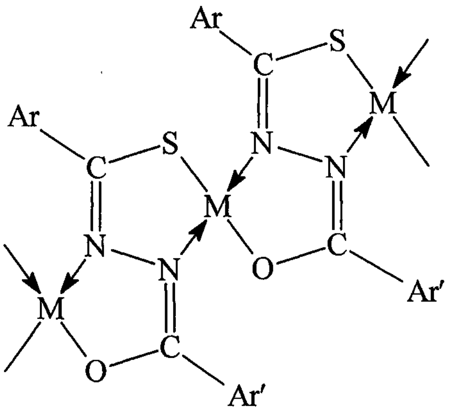

[M(Iotbh)] M = Cu(II), Zn(II) and Ni(II)

$Ar = 2\text{-}(HO)C_6H_4$, $Ar' = 4\text{-}C_5H_4N$

**Fig. 1:** Proposed structure of the complexes.

is further supported by the presence of new bands at 1597 and 837 $cm^{-1}$ for ν(C=N) of NCO and ν(C-S), respectively. Thus, the ligand binds Mn(III) and Fe(III) through enolic oxygen, thiolato sulfur and phenolate oxygen in addition to one hydrazine nitrogen. The positive shifts of 42 and 20 $cm^{-1}$ in thioamide I and ν(N-N), respectively, also suggest the involvement of these groups in bonding. Furthermore, the presence of bands due to ν(OH) and $\rho_r(H_2O)$ suggest the coordinated nature of water molecules.

### Mössbauer spectra

The Mössbauer spectrum (Fig. 2a) of $[Fe(Iotbh\text{-}H)(H_2O)_2]$ at room temperature shows a singlet with isomer shift of $\delta = 0.32$ mm $s^{-1}$ and quadrupole splitting of $\Delta = 0.31$ mm $s^{-1}$ typical of high-spin iron(III). On lowering the temperature to 78 K it shows (Fig. 2b) the presence of two sites. The values of isomer shift and quadrupole splitting for site 1 are 0.25 and 0.48 mm $s^{-1}$, respectively, for high-spin iron(III) and the corresponding MB parameters for site 2 are 0.68 and 0.84 mm $s^{-1}$, respectively, for low-spin iron(III) in 3:1 ratio, thereby, suggesting the presence of both high-spin and low-spin states of iron(III) at 78 K.

### ESR spectra

[Cu(Iotbh)] shows an isotropic ESR spectrum at room temperature. The $g_{iso} = 2.086$ is typical of a Cu(II) chelate ascribing a square-planar geometry around the metal ion. The room temperature solid state ESR spectrum of $[Fe(Iotbh\text{-}H)(H_2O)_2]$ exhibits $g_\perp = 5.654$ and $g_\| = 2.042$ arising from very large zero-field splitting in six-coordinate, high-spin iron(III). The values of $g_\perp$ and $g_{||}$ also suggest distorted octahedral geometry around Fe(III) /20/.

### Antitumor Studies

To investigate the antitumor activity of $H_2$Iotbh and its metal complexes, their effect on the growth of Dalton's Lymphoma (DL) cells *in vitro* was measured by MTT assay. It was observed that treatment of tumor cells with a DMSO solution of [Cu(Iotbh)] caused maximum growth inhibition followed by $[Fe(Iotbh\text{-}H)(H_2O)_2]$ and the ligand (Table 2). The reason for the observed inhibition of tumor cell growth by the metal complexes is unclear, however, several possibilities could be considered. The cytostatic activity of the metal complexes could be a direct result of the interaction of the metal complexes with DNA, thus interfering with the process of DNA replication. Indeed, DNA binding of metal complexes has been documented /21/. Furthermore, inhibition/activation of various enzymes directly/indirectly involved in DNA replication is not ruled out. Interaction of metal complexes with protein components of viable cells has been reported /22,23/. Binding of metal complexes with protein may cause alterations in the structural and functional organization of proteins. However, more studies will be needed to confirm the existence of one or more of such possibilities in our system. These results may also indicate a possible decline of the overall metabolic activity of the tumor cells with a concomitant inhibition of the activity of various enzymes involved in respiration.

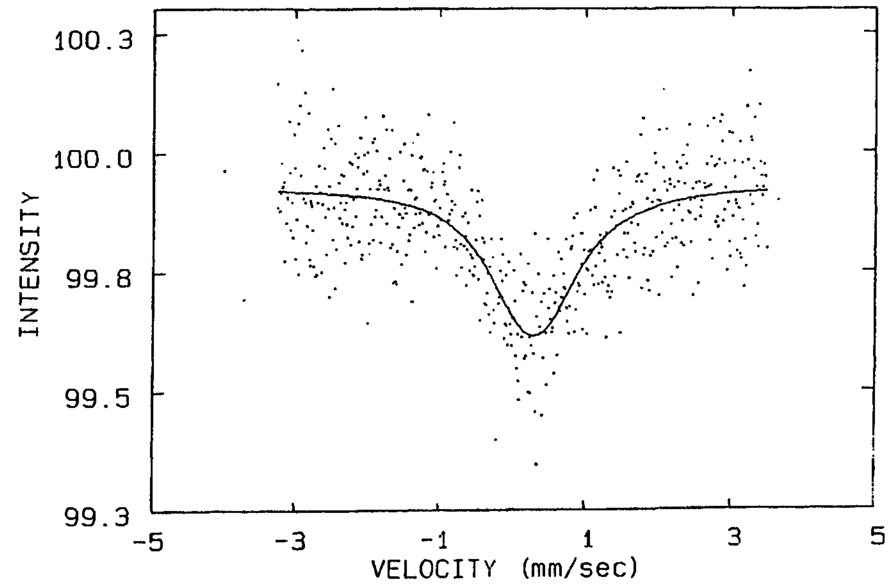

(a)

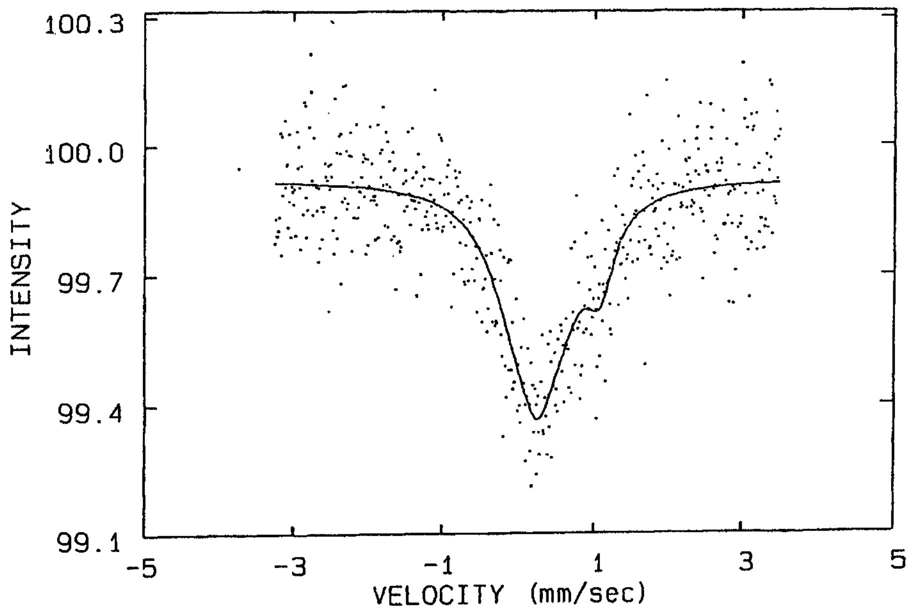

(b)

**Fig. 2:** (a) Mössbauer spectrum of [Fe(Iotbh-H)($H_2O_2$] at room temperature; (b) Mössbauer spectrum of [Fe(Iotbh-H)($H_2O_2$] at 78 K.

**Table 2**

Effect of $H_2$Iotbh and its metal complexes on tumor cell growth *in vitro* ($IC_{50}$ values in μg/mL).

| Compound | $IC_{50}$ (μg/mL) |
|---|---|
| $H_2$Iotbh | 26.40 |
| [Fe(Iotbh-H)($H_2O$)$_2$] | 14.79 |
| [Cu(Iotbh)] | 0.97 |
| *Cisplatin* | 0.71 |

$IC_{50}$ = average drug concentration (μg/mL in DMSO) for 50% inhibition of tumor cells growth. Values are mean ± SD of three experiments. $p < 0.05$ with respect to values of $IC_{50}$ of ligand alone.

Although the metal complexes showed cytostatic effects on the tumor cells *in vitro*, these results do not necessarily indicate if these cells are actually killed by the direct action of the metal complexes. To check this, in the next part of the investigation we studied the effect of the metal complexes on the tumor cell killing with respect to the mode of cell death. Cisplatin has been reported to induce apoptotic cell death in the tumor cells /24,25/. It was observed that the tumor cells were killed by the induction of apoptosis (Fig. 3a). [Cu(Iotbh)] was found to be most effective in the induction of tumor cell apoptosis. The mechanism of the induction of apoptosis remains poorly understood and is thought to be dependent on multiple mechanism(s) ultimately culminating in the activation of DNA cleaving endonucleases /24,26/. As shown in Fig. 3b, [Cu(Iotbh)] and [Fe(Iotbh-H)($H_2O$)$_2$] cause an increase in % specific DNA fragmentation; it is probable that these metal complexes may act via the activation of endonucleases to kill the tumor cells by inducing apoptotic cell death.

Furthermore, the metal complexes at the concentration checked did not inhibit the growth of normal splenocyte and bone marrow cells (data not shown), which comprise a major proportion of proliferating cells indicating that the cytostatic effect of the metal complexes was limited to the tumor phenotype and not on all proliferating cells. The killing of tumor cells specifically could be attributed to the susceptibility of tumor cell DNA to a damage by metal complexes as compared to the normal cells. Indeed, in the case of cisplatin it has been shown that the tumor cells are killed specifically because they are unable to overcome the genetic load of mutations caused by the drug because of their defective DNA repair mechanism /27/.

In the next part of the investigation we checked the life prolonging effect in DL bearing mice administered with PBS (phosphate buffer saline) alone or containing the ligand or metal complexes as indicated in the experimental section. As shown in Table 3 minimal % T/C was observed in mice administered with the ligand alone as compared to that of mice administered with metal complexes. Maximum % T/C was found for [Cu(Iotbh)] followed by that of [Fe(Iotbh-H)($H_2O$)$_2$]. Our observations show that $H_2$Iotbh and [Zn(Iotbh)] did not have antitumor activity whereas, significantly higher life prolonging ability in tumor bearing mice were observed for [Cu(Iotbh)] and [Fe(Iotbh-H)($H_2O$)$_2$]. Increase in the value

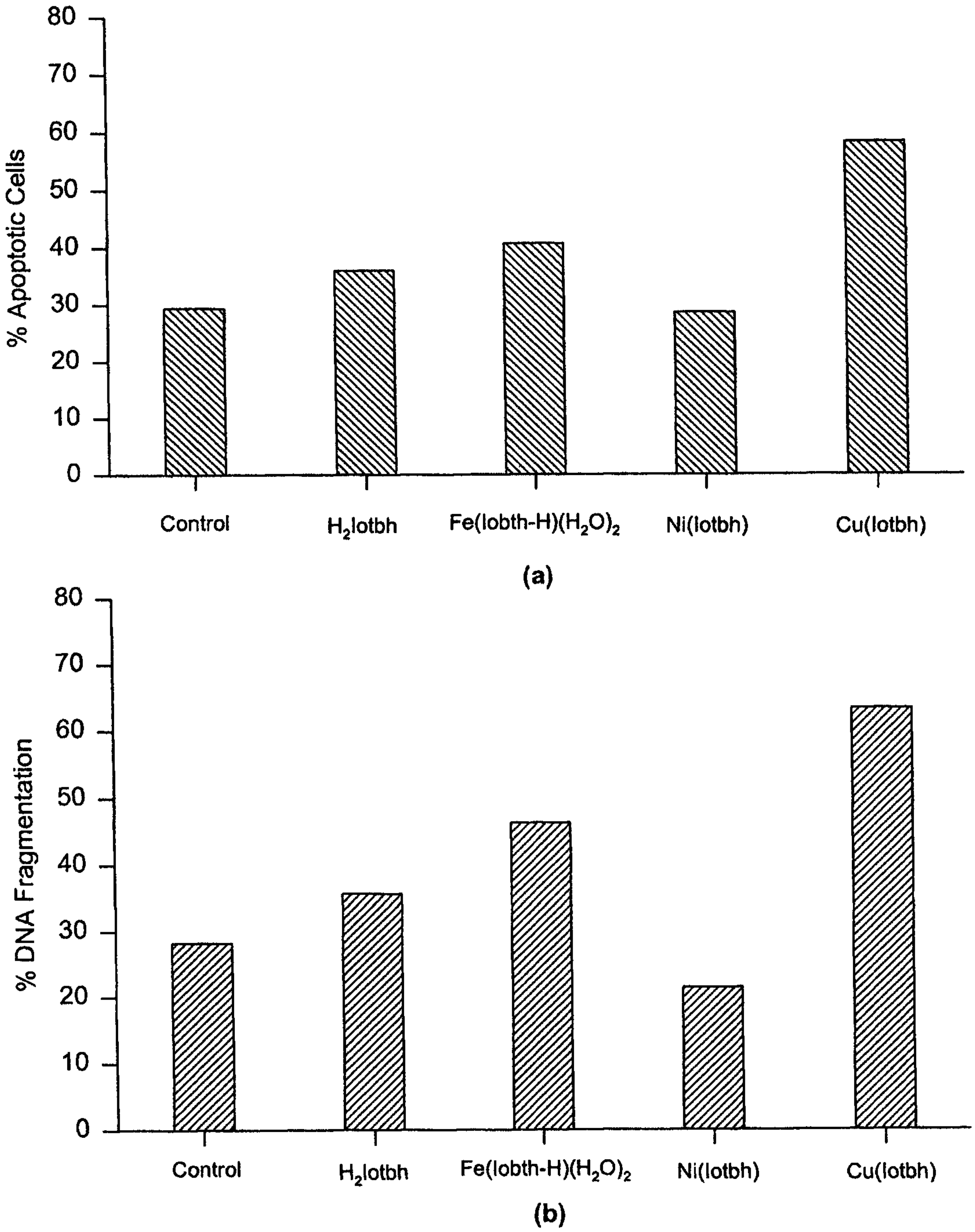

**Fig. 3:** (a) Effect of ligand and its metal complexes on the induction of apoptosis in DL cells. Cells were incubated in medium alone or containing ligand or its metal complexes (10 μg/mL) for 24 h and the number of cells showing apoptotic morphology was enumerated. Values are mean of three experiments. (b) Effect of ligand and its metal complexes on % DNA fragmentation of tumor cells. DL cells were incubated in medium alone or containing ligand or its metal complexes (10 μg/mL) for 24 h and the % DNA fragmentation was evaluated. Values are mean of three experiments.

of % T/C, an indicator of the effect of drug administration on the survival of tumor bearing mice, suggests that such an effect could result either from the direct cytotoxic/cytostatic action of the complexes on tumor cells or from the activation of certain host derived mechanism resulting in a decrease of tumor load.

**Table 3**

Effect of *in vivo* administration of $H_2$Iotbh and its metal complexes on the survival of tumor bearing mice.

| Compound | Post inoculation life span (% T/C) |
|---|---|
| $H_2$Iotbh | 140 |
| [Zn(Iotbh)($H_2O$)$_2$] | 150 |
| [Fe(Iotbh-H)($H_2O$)$_2$] | 210 |
| [Cu(Iotbh)] | 220 |
| *Cisplatin* | 250 |

Treatment responses (six mice per treatment group) presented as % T/C, was calculated according to the equation: mean life span of treated mice/mean life of control mice by 100. A % T/C $\geq$ 125 is considered biologically significant. C = 20 $\pm$ 2 days, experiments terminated after 50 days. Values are mean $\pm$ SD of the three experiments. $\rho < 0.05$ with respect to values of $H_2$Iotbh alone.

Progression of growth of various tumors including DL is invariably associated with the onset of immunosuppression in tumor bearing host /28-31/, one of the reasons being induction of apoptosis and inhibition of proliferation of hematopoietic precursor cells /32,33/. Since *in vivo* administration of these metal complexes prolonged survival of tumor bearing animals and these metal complexes did not show cytotoxicity against normal cells *in vitro*, we were interested to investigate if the administration of metal complexes could reverse tumor growth associated induction of apoptosis in various hematopoietic cells. For this DL bearing mice were administered with metal complexes, and the % of apoptotic thymocyte, splenocyte and bone marrow cells were enumerated. As shown in Fig. 4a, administration of metal complexes in tumor bearing mice resulted in the inhibition of tumor associated apoptosis of thymocyte, splenocyte and bone marrow cells. Similar results were obtained for % DNA fragmentation as well (Fig. 4b). The reversal of tumor growth associated induction of apoptosis of hematopoietic cells by metal complexes is predicted to be due to two reasons: 1. reduction of tumor load resulting due to the cytotoxic effect of metal complexes on tumor cells, leading to a decrease in the tumor associated concentration of apoptotic factors. 2. direct protective effect of metal complexes on the hematopoietic cells. Although not very clear, the probability of the latter could be due to the fact that metal complexes can bind to DNA and several proteins in cells, which could result in the protective effect.

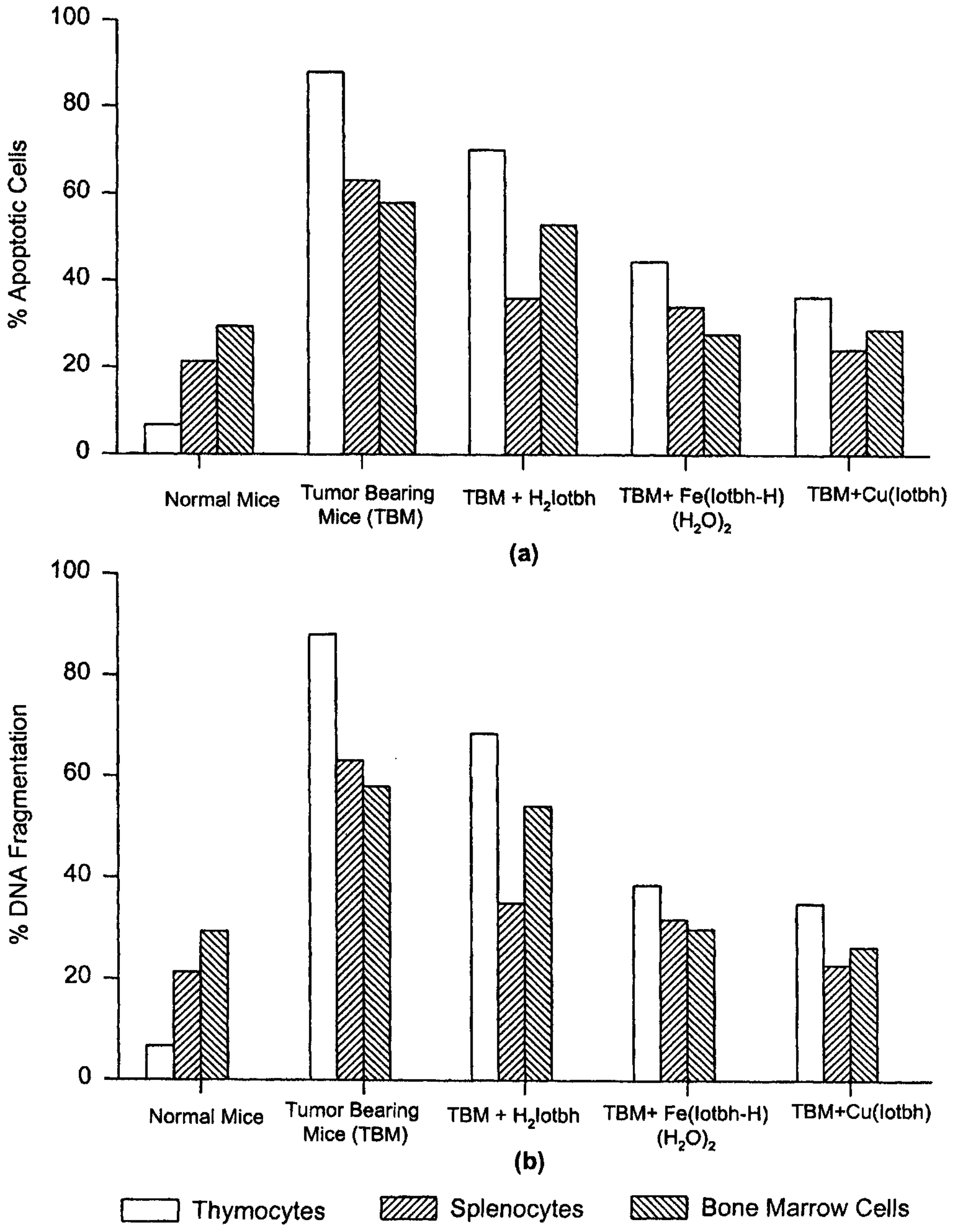

**Fig. 4:** Effect of *in vitro* administration of ligand or its metal complexes on the induction of apoptosis in thymocytes, splenocytes and bone marrow cells of normal or DL-bearing mice treated with ligand or its metal complexes. Thymocytes, splenocytes and bone marrow cells of normal or tumor-bearing mice were treated with ligand or its metal complexes and the apoptotic cells were enumerated. Values are mean of three experiments. (b) Effect of *in vivo* administration of ligand or its metal complexes on % DNA fragmentation of thymocyte, splenocyte and bone marrow cells of normal or tumor-bearing mice with ligand or its metal complexes. Thymocytes, splenocytes and bone marrow cells of normal or DL-bearing mice were treated with ligand or metal complexes and checked for % DNA fragmentation. Values are mean of three experiments.

Although more investigations will be required to confirm the mechanism of action of metal complexes on tumor and normal cells, the study suggests that [Cu(Iotbh)] and [Fe(Iotbh-H)($H_2O$)$_2$] can cause prolongation of survival in tumor bearing animals by:

1. Directly killing tumor cells.
2. Reversing tumor associated immunosuppression.

The finding of this investigation may have long lasting clinical implications with the novel proposition that metal complexes of N-isonicotinoyl- N'-*o*-hydroxythiobenzhydrazide may have a dual mechanism of action in tumor regression.

### EXPERIMENTAL

### Physical measurements

The complexes were analysed for their metal and sulfur contents as described elsewhere /34/. Carbon, hydrogen and nitrogen contents were estimated on a Perkin-Elmer 240C microanalyzer. The infrared spectra of the ligand and its complexes were recorded on a JASCO FT/IR-5300 spectrophotometer in KBr in the 4000-400 $cm^{-1}$ region. The electronic spectra were recorded on a Cary-2390 UV-Visible spectrophotometer in DMSO solution and as Nujol mulls /35/. Room temperature magnetic susceptibility measurements were made on a Cahn Faraday electrobalance using cobalt mercury tetrathiocyanate as a calibrant and the experimental magnetic susceptibilities were corrected for diamagnetism by using the procedure of Figgis and Lewis /36/. The Mössbauer spectra were collected using a Cryophysics MS-1 microprocessor controlled spectrometer operating in the constant acceleration mode.

### Antitumor screening

#### *Mice*

Inbred populations of BALB/c mice of either sex of 8-12 weeks were used for the study. The mice were fed food and water *ad libitum* under pathogen-free conditions and were treated with utmost human care.

#### *Tumor Systems*

Dalton's Lymphoma (a spontaneous murine T cell lymphoma) were maintained in culture *in vitro* as well as in ascites by serial transplantation in BALB/c mice by an intraperitoneal injection of $5\times10^5$ cell/mouse.

#### *Thymocyte preparation and culture*

Thymuses obtained from normal and tumor bearing mice with or without administration of the DMSO solutions of the complexes were weighed on a chilled watch glass, diced on ice and passed through a stainless steel screen using a syringe plunger. These cells, after washing with phosphate buffered saline (PBS) by centrifugation at 200g for 10 min at 4 °C, were used directly for thymocyte counts. Cell viability in the thymocyte preparation was determined by mixing 10 mL sample with an equal volume of 0.4% trypan blue-PBS solution /37/ and counting the cells on a hemocytometer under light microscope. Cells that did not exclude trypan blue were considered nonviable. For culturing thymocytes *in vitro*, thymocytes were maintained in complete RPMI 1640 medium at 37 °C in a humidified atmosphere of 5% $CO_2$ in air.

***Splenocyte Preparation and Culture***

Spleens obtained from normal and tumor bearing mice with or without administration of complexes were weighed on a chilled watch glass, diced on ice and passed through a stainless steel screen using a syringe plunger. These cells were washed with phosphate buffered saline (PBS) by centrifugation at 200x g for 10 min at 4°C, and erythrocytes were then depleted by treatment with 0.84% ammonium chloride for 10 min at room temperature. Cells were again washed in PBS and then cultured in a humidified atmosphere at 5% $CO_2$, to remove adherent cells. Non-adherent cells were collected and used for assay.

***Bone Marrow Cell Preparation and Culture***

Bone marrow cells (BMC) were obtained from the femurs of normal and tumor bearing mice with or without administration of complexes, as described elsewhere /38/. Briefly, mice were killed by cervical dislocation and the BMC were obtained from the femoral shafts by flushing them with serum-free medium. BMC were then agitated gently to prepare a single cell suspension and then washed thrice with serum-free medium by centrifugation at 200x g at 4 °C. BMC were then incubated in plastic tissue culture flask for 2 h at 37 °C to remove the adherent macrophage. The non-adherent BMC were then used for proliferation.

***Proliferation assay***

Different cells obtained from normal or tumor bearing mice treated with or without complexes were incubated at a concentration of 1.5 x $10^6$ cells per well in a 96 well plastic tissue culture plate with medium containing sub-mitogenic doses of concanavalin-A (1 μg/mL). Cultures were then incubated at 37 °C in a $CO_2$ incubator for 48h and assayed for proliferation and growth inhibition using MTT assay.

***In vitro growth inhibitory assay***

The MTT assay was used to measure the cytotoxic effect of the ligand and the complexes. The procedures employed the pale yellow tetrazolium salt [3-(4,5-dimethylthiazol)-2-yl-2,5-diphenyl-2H-tetrazolium bromide] (MTT), which was cleaved by active mitochondria to form a dark blue formazan product that can be completely solubilized in acidic isopropanol /39/. The assay provides a simple way to detect living and growing cells without use of radioactivity. Briefly, 5 × $10^4$ tumor cells were plated in triplicate in a 96-well flat bottom tissue culture plates, and treated with different concentrations of drugs for the time indicated. MTT (0.005 g $cm^{-3}$ in PBS) was added to the cell culture and incubated for 4h at 37 °C in a 5% $CO_2$ humidified incubator. The formazan crystals formed during the reaction were dissolved in 100 μL of 0.04N HCl in isopropanol and absorbance was read at 570 nm. The average drug concentration (μg/mL) for 50% inhibition ($IC_{50}$) of tumor cell-growth was determined by plotting the log of drug concentration *versus* the growth rate (% control).

***Morphological evaluation of apoptotic cells***

Cells were air dried, fixed in methanol, stained with Wright staining solution, mounted in glycerine and analyzed under light microscope at 45x magnification. Apoptotic cells were identified on the basis of morphological features that included contracted cell bodies, condensed, uniformly circumscribed and densely stained chromatin, or membrane-bound apoptotic bodies containing one or more nuclear fragments /40/. The percentage of apoptotic cells was determined by counting more than 300 cells in at least three separate visions.

***Quantitation of percent DNA fragmentation***

Percent DNA fragmentation was quantified following a method described by Sellins and Cohen /41/ with slight modification. Cells (5 x $10^5$ cells/mL) were suspended in 0.5 mL of lysis buffer (Tris-EDTA buffer, pH 7.4 containing 0.2% Triton X-100) and centrifuged for 15 min at 13000x g at 4 °C in a microfuge tube (labeled as B). Supernatant was transferred to another tube (labeled as T). 0.5 mL of 25% trichloroacetic acid was added to T and B tubes, which were then to be vortexed vigorously. Tubes were kept overnight at 4 °C for precipitation. Supernatant was discarded after centrifugation at 13000x g for 10 min and then DNA in each pellet were hydrolyzed with 80 µL of 5% trichloroacetic acid by heating on water bath at 90 °C for 15 min and 160 µL of freshly prepared diphenylamine (150 mg diphenylamine in 10 mL glacial acetic acid, 150 µL conc.$H_2SO_4$ and 50 mL of acetaldehyde solution) was added and the tubes were allowed to stand overnight at room temperature to develop colour. 100 µL of this coloured solution was transferred to a 96 well flat bottom ELISA plate and absorbance at 600 nm noted on an ELISA plate reader. Percent fragmented DNA was calculated using the formula:

$$\%\ \text{fragmented DNA} = \frac{T}{T+B} \times 100$$

where T = absorbance of fragmented DNA, T + B = absorbance of total DNA.

***In vivo studies***

In order to assess the antitumor activity of the compounds, 6-8 groups of BALB/c mice were inoculated intraperitoneally with DL cells ($10^6$) followed by treatment with the metal complexes (10 mg /kg body weight) in a single i.p. injection on days 1, 5, 9 and 12 after tumor transplantation. This treatment protocol was selected for administration, as previous studies /42/ have shown that metal complexes of sulfur donor ligands have shown optimal antitumor activity at a dose of 10 mg/kg. The antitumor efficacy of each agent is expressed as % T/C and is given by,

$$\%\ T/C = \frac{\text{Mean life span of treated mice}}{\text{Mean life span of control mice}} \times 100$$

### MATERIALS AND METHODS

Sodium salt of *o*-hydroxydithiobenzoate: A solution of *o*-hydroxybenzaldehyde (21mL ~ 25g) in 60 mL of ethanol was heated to 65 °C and 135 mL of the filtered ammonium polysulfide solution was added in 10 mL portions during 10 min., keeping the temperature at 65°C. The reaction mixture was boiled to reflux for 1 h, immediately cooled in ice, 200 mL of ether was added and the solution was acidified with conc. HCl. The dithioacid that separated as red oil was filtered through suction to remove the precipitated sulfur. The filtrate was transferred into a separating funnel, 100 mL of ether was also added and the ethereal layer containing the dithioacid was separated and washed with cold distilled water. The red coloured sodium salt of the dithioacid was extracted by shaking the ethereal solution of the dithioacid twice with aqueous NaOH solution(8g in 100 mL).

***Carboxymethyl-o-hydroxydithiobenzoate:*** To the sodium salt of *o*-hydroxydithiobenzoate was added a solution of chloroacetic acid (20g) neutralized with sodium carbonate and pH of the mixture was adjusted to ~7. After standing the reaction mixture overnight at room temperature, the dark solution was acidified with conc. HCl and the ester which separated on cooling was filtered off, washed with cold water and recrystallized from hot ethanol in the presence of animal charcoal, m.p. 120°C (lit. 121°C) /43/.

***N-Isonicotinoyl-N'-o-hydroxythiobenzhydrazide ($H_2$Iotbh)*** was prepared by mixing an equivalent quantity of carboxymethyl-*o*-hydroxydithiobenzoate and isonicotinic acid hydrazide, each dissolved separately in one equivalent of 1N NaOH solution and adding acetic acid dropwise to the above ice cooled solution, after standing the solution for ~2 h. The product thus obtained was suction filtered, washed with water and recrystallized from ethanol which yielded white compound. [Co(Iotbh)($H_2O$)$_2$], [M(Iotbh)] [M = Ni(II), Cu(II) or Zn(II)] and [M(Iotbh-H)($H_2O$)$_2$] [M = Mn(III) or Fe(III)] were prepared by adding a DMF solution (10 mL) of $H_2$Iotbh (0.8g, 2.9 mmol) dropwise to the ethanolic/aqueous solution (20 mL) of the respective metal(II) acetate or $FeSO_4.7H_2O$ in an 1:1 molar ratio. Coloured precipitate of the complexes started separating out after 5 min of stirring at room temperature but the reaction mixture was further stirred at room temperature for 2 h to ensure the completion of the reaction except for the Mn(III) where, there was no precipitation even after 3 h of stirring. The reaction mixture changed from wine red to maroon after 15 min of stirring. The Mn(III) complex was precipitated by the addition of acetonitrile. The insoluble complexes thus obtained were filtered off, washed with ethanol and dried in *vacuo*. The products obtained were characterized by various physico-chemical methods.

### REFERENCES

1. M.A. Ali and S.E. Livingstone, *Coord. Chem. Rev.*, **13**, 101 (1974).
2. N.K. Singh, S. Agrawal and R.C. Aggarwal, *Polyhedron*, **3**, 1271 (1984).
3. N.K. Singh, S. Agrawal and R.C. Aggarwal, *Synth. React. Inorg. Met.-Org. Chem.*, **15**, 75 (1985).

4. S. Agarwal and N.K. Singh, *Spectrochim. Acta*, **42**, 507 (1986).
5. N.K. Singh, S. Agarwal and R.C. Aggarwal, *Ind. J. Chem. (A)*, **23**, (1984) and ref. therein
6. K.A. Jensen and J.F. Miquel, *Acta Chem. Scand.*, **6**, 189 (1952).
7. H.A. Offe, W. Siefken, G. Domag, G.B. Baker and W.G. Taylor, *Can. J. Pharm. Sci.*, **7**, 100 (1972).
8. N. Nawar and N.M. Hosny, *Chem. Pharm. Bull.*, **47**, 944 (1999).
9. N. Nawar and N.M. Hosny, *Transition Met. Chem.*, **25**, 1 (2000).
10. E.W. Ainscough, A.M. Brodie, W.A. Deny, G.J. Finlay, S.A. Goche and J.D. Ranford, *J. Inorg. Biochem.*, **77**, 125 (1999).
11. B. Swamy and J.R. Swamy, *Transition Met. Chem.*, **16**, 35 (1991).
12. D.B. Young and K. Duncan, *Ann. Rev. Microbiol.*, **49**, 641 (1995).
13. H.J. Smith and D.R. Williams, *Introduction to Principles of Drug Design and Action*, 3rd Ed. Harwood Academic Press, 1998, p. 528.
14. S. Kakimoto and K. Yamamoto, *Pharm. Bull.*, **4**, 4 (1956).
15. N.K. Singh, S. Agarwal and R.C. Aggarwal, *Ind. J. Chem. (A)*, **24**, 617 (1984).
16. S. Agarwal, N.K. Singh, R.C. Aggarwal, A. Sodhi, and P. Tandon, *J. Med. Chem.*, **29**, 199 (1986).
17. N.K. Singh, U. Sharma and S. Agarwal, *Transition Met. Chem.*, **13**, 233 (1988).
18. N.K. Singh, N. Singh, G.C. Prasad, A. Sodhi and A. Shrivastava, *Bioorg. Med. Chem.*, **5**, 245 (1997).
19. K. Geetharani and D.N. Sathyanarayana, *Austr. J. Chem.*, **30**, 1617 (1977).
20. B.A. Goodman and J.B. Raynor, *Adv. Inorg. & Radio Chem.*, Academic Press, New York, 1970, Vol.13, p.135.
21. S. Steinkopf, A. Garoufis, W. Nerdal and E. Sletten, *Acta Chem. Scand.*, **49**, 495 (1995).
22. R.K. Watt and P.W. Ludden, *Cell Mol. Life Sci.*, **56**, 604 (1999).
23. N.F. Krynetskaya, E.A. Kubareva, M.A. Timchenko, V.M. Belkov and Z.A. Shabarova, *Biochemistry (Mosc)*, **63**, 1068 (1998).
24. P. Ranjan, A. Sodhi and S.M. Singh, *Anticancer Drugs*, **9**, 333 (1998).
25. A. Eastmen, *Cancer Cells*, **2**, 275 (1990).
26. T.M. Buttke and P.A. Sandstrom, *Immunol. Today*, **15**, 7 (1994).
27. B. Rosenberg, *Cancer*, **55**, 2303 (1985).
28. S.M. Singh, P. Parajuli, A. Srivastava and A. Sodhi, *Int. J. Immunopathol. Pharmacol.*, **1**, 27 (1997).
29. P.J. Deckers, R.C. Davis, G.A. Parker and J.A. Mannick, *Cancer Res.* **33**, 33 (1973).
30. C.M. Loeffler, M.J. Smyth, D.L. Longo, W.C. Kopp, L.K. Harvey, H.R. Tribble, J.E. Tase, W.J. Urba, A.S. Leonard, H.A. Young and C. Ochoa, *J. Immunol.*, **149**, 949 (1973).
31. D. Salitzeanu, *Adv. Cancer Res.* **60**, 246 (1990).
32. C.C. Handy and L. Balducci, *Amer J. Med. Sci.*, **290**, 196 (1985).
33. A. Kumar and S.M. Singh, *Immunol. Cell Biol.*, **73**, 220 (1995).
34. A. Shrivastav, N.K. Singh and S.M. Singh, *Bioorg. Med. Chem.*, **10**, 887 (2002).
35. R.H. Lee, G. Griswold and J. Kleinberg, *Inorg. Chem.*, **3**, 1278 (1964).
36. B. N. Figgis and J. Lewis, *Modern Coordination Chemistry*; J. Lewis, R.G. Wilkins, Eds.; *Interscience*, New York, 1960, p. 403

37. A. Shanker and S.M. Singh, *Tumor Biol.*, 591 (2000).
38. P. Parajuli, S.M. Singh and A. Kumar, *Int. J. Immunopharmac.*, **17**, 1 (1995).
39. A. Sanker and S.M. Singh, *Neoplasma*, **47**, 2 (2000).
40. J.F.R. Kerr, A.H. Wyllie and A.R. Currie, *Br. J. Cancer*, **26**, 239 (1972).
41. K.S. Sellins and J.J. Cohen, *J. Immunol.*, **139**, 3199 (1987).
42. N.K. Singh, A. Srivastava, P. Ranjan and A. Sodhi, *Transition Met. Chem.*, **25**, 133 (2000).
43. K.A. Jensen and C. Pedersen, *Acta Chem. Scand.*, **15**, 1087 (1961).

